# Editorial: Advances in proctology and colorectal surgery

**DOI:** 10.3389/fsurg.2023.1344739

**Published:** 2023-12-11

**Authors:** Marta Goglia, Mario Trompetto, Alberto Realis Luc, Giuseppe Clerico, Gaetano Gallo

**Affiliations:** ^1^ Department of Medical and Surgical Sciences and Translational Medicine, Faculty of Medicine and Psychology, School in Translational Medicine and Oncology, Sapienza University of Rome, Rome, Italy; ^2^ Department of Colorectal Surgery, S. Rita Clinic, Vercelli, Italy; ^3^ Department of Surgery, Sapienza University of Rome, Rome, Italy

**Keywords:** colorectal surgery, proctology, pelvic floor, functional diseases, hemorrhoids, pilonidal disease, inflammatory bowel disease, rectal cancer

**Editorial on the Research Topic** Advances in proctology and colorectal surgery

Proctology and colorectal surgery for benign disorders and neoplasia represent a broad field of general surgery. Their relevance is well known, on the one hand because of the still extremely high frequency in Western countries of colon-rectal cancer (currently estimated as the third most frequent cancer in the world), and on the other hand because of the serious impact on the quality of life (QoL) of patients suffering from benign, inflammatory, and functional diseases of the lower gastrointestinal tract. This Research Topic of Frontiers in Surgery, composed by forty-five original articles on colorectal surgery and proctology addresses several topics including non-surgical solutions, diagnostic aspects, translational research, and specific scenarios.

Rectal cancer, which accounts for about 30% of all colorectal malignancies, has been studied from several perspectives. The routine use of the LARS score after rectal surgery to assess the bowel function and QoL of patients is highly recommended (De Simone et al.).

Pacevicius et al. conducted a case-control study to investigate the differences in terms of overall survival and surgical outcome between the invasive surgical approach with TME and local excision (LE) ± chemotherapy of early rectal cancer. The authors identified that approximately 85.2% of the patients had no Low Anterior Rectal Resection Syndrome (LARS) in LE group compared with 54.5% in TME group (*p* = 0.018); furthermore, they reported comparable survival outcomes in the two groups, thus favoring a less invasive surgical approach in early stages such as LE for better QoL outcomes. Herzberg et al. as well investigated the QoL in terms of LARS in patients who underwent rectal resection and End-to End primary anastomosis in favor of the first one using a standardized perioperative pathway. An interesting radiological study was conducted using innovative imaging techniques to correlate the oncological outcome to inadvertent residual pelvic diaphragm on postoperative MRI after extralevator abdominoperineal excision (ELAPE) or the conventional abdominoperineal excision (c-APE) demonstrating that anterior tumor orientation was a risk factor for circumferential resection margin (CRM) involvement regardless of surgical approach (Oerskov et al.). Tumour downsizing of rectal cancer to allow a R0 resectablility is a long-standing problem, especially for those patients who do not tolerate neoadjuvant conventional chemoradiation (CRT). A research group from Germany proposes short-term neoadjuvant radiotherapy (5 × 5 Gy) followed by an interval before surgery (SRT- delay) as a valid alternative to CRT with comparable results in those patients who cannot tolerate CRT (Albrecht et al.).

In this regard of personalized medicine, Coletta et al. published a state of the art focused on the de-functioning ileostomy techniques highlighting the relevance of a tailored surgical approach in those patients.

In fact, this editorial has given numerous authors the opportunity to highlight and report unique cases in lower GI abdominal surgery in which the patient-specific surgical approach has demonstrated successful results (Zhang et al.).

Concerning colon cancer surgery, a current debate on the management of synchronous and metachronous metastasis is ongoing. The selection of a surgical approach for liver resection (SLR) should take into account various factors, including the tumor's location, size, and resectability, the overall health of the patient (including age, comorbidities, and prior treatments), and the surgeon's experience. SLR represents a safe and effective option for patients with primary liver metastases of limited extent. It offers advantages such as reduced intraoperative bleeding, quicker recovery of intestinal function, shorter postoperative hospital stays, and lower rates of surgical complications compared to open laparotomy. Importantly, there are no significant differences in long-term outcomes between the two approaches. It is worth noting that there is currently insufficient high-quality evidence to establish the superiority of one approach over the other. Therefore, future studies should involve larger patient cohorts and randomized controlled trials to provide more definitive insights into the most appropriate strategy (Sena et al.). Also, synchronous liver resection (LR), cytoreductive surgery (CRS), and hyperthermic intraperitoneal chemotherapy for colorectal liver and peritoneal metastases have been investigated. However, the role of combined surgical strategy extensive surgical approach including CRS with hyperthermic intraperitoneal chemotherapy (HIPEC) and LR is still controversial. (Di Carlo et al.)

The standardized surgical procedure techniques are also evolving and being studied towards modern surgery in continuous progress. For example, Xu et al. studied a novel knotless hand-sewn end-to-end anastomosis using V-loc barbed suture vs. stapled anastomosis in laparoscopic left colonic surgery and demonstrated that this technique can reduce operating time and costs for the hospital when compared to the technique using staplers. The authors therefore propose it as a safe and feasible technique. Anastomotic leak prevention has also been investigated by the group of Baeza-Murcia with a propensity score-matched study on bowel mechanical preparation and oral antibiotics use. They confirmed that oral antibiotics, mechanical bowel preparation and inflammatory markers, significantly reduces morbidity adjusted to severity of complications, the anastomotic leakage rate, hospital stay and readmissions (Baeza-Murcia et al.). A multicenter study by Admasu et al. instead, demonstrated the need to increase the level of alertness regarding blood disorders such as coagulopathy even in the presence of colorectal polyps with a prevalence of 76 (50.7%; 95% CI: 45.66, 54.34). As well as associations of advanced age with comorbidity, stage and primary subsite as contributors to mortality from colorectal cancer are currently still in force (Gheybi et al.).

Moreover, the use of alternative procedures in the postoperative period that can prevent postoperative complications and promote the recovery of patients after major abdominal surgery is also making incredible progress. A recent paper by Zhao et al. investigated the effects of acupuncture and electroacupuncture in postoperative ileus prevention with promising results with shorter time to the first flatus [stand mean difference (SMD), −0.57; 95% CI, −0.73 to −0.41, *p* < 0.00001], shorter time to the first defecation [mean difference (MD), −4.92 h, 95% CI −8.10 to −1.74 h, *p* = 0.002] than the control group.

Another super topical subject in colorectal surgery is the use of indocyanine green, not only for the evaluation of tissue perfusion but also for the assessment of the risk of anastomotic dehiscence. Image guided surgery in fact represents the most modern frontier of technology development in surgery. Maione et al. were able to demonstrate that the intraoperative use of Near-Infrared Fluorescence-Indocyanine Green in colorectal surgery is safe, feasible, and associated with a substantial reduction in postoperative anastomotic leakage rate. As well as, ICG has shown promising results as a safe and reproducible technique for the preoperative tumour marking prior of robotic resections (Konstantinidis et al.).

In addition, some less frequent but complex medical and hospital management pathologies, with a huge impact on the patient's life and with huge consequences also on the inpatient ward in terms of nursing support, complications, costs for the company, are also the responsibility of the emergency surgeon and have been analyzed in this editorial. For example, Fournier's gangrene is a pathology that places the patient's life at a very high risk and whose only treatment at present is surgical debridement with great loss of tissue associated with antibiotic therapy with the need for long hospitalization. Tutino et al. retrospectively analyzed a series of cases in which the hyperbolic chamber was used in support of surgical therapy to see whether hyperbolic therapy was associated with a better prognosis. The authors showed, however, that the hospital stay was longer in patients treated with hyperbaric oxygen therapy [mean 11 (C.I. 0.50–21.89) vs. mean 25 (C.I. 18.02–31.97); *p* = 0.02] without an improvement in survival (*p* = 1.00), while the delay in treatment was associated with a higher risk of mortality in their case series. Another peculiar and rare pathological condition that has to be known form is the diagnosis of rectal cancer in a patient symptomatic for rectal prolapse (Jurić et al.). Similarly, also appendiceal tumors represent a rare but relevant incidental finding (0.5%) after appendicectomy that challenge the physicians that deserves further investigations starting from the work of Viel et al.

Likewise, certain measures in clinical practice have been identified and proven effective. For example, the recently published propensity score by Jiang et al. stated regarding surgical infections that the clichéd incisional press after suturing is a simple, costless, and effective intervention in reducing superficial incisional SSI. Furthermore, the current knowledge on C. difficile infection after colorectal surgery was also re-evaluated, confirming that fresh faecal bacteria are the best treatment, but frozen and freeze-dried faecal bacteria can achieve the same effect (Yang et al.). Another extremely frequent condition routinely dealt with by the emergency surgeon is incarcerated inguinal hernia for which Chen et al. proposed a predictive model of bowel resection based on the systemic immune-inflammation index. Indeed, they demonstrated that the increased risk of bowel resection is highly correlated among the elderly (≥70 years) and for persons with elevated temperature (≥37.3°C), high systemic immuno-inflammation index (SII) values (≥1,230.13), presence of bowel obstruction, and signs of peritonitis.

The Covid-19 pandemic has posed a great challenge for medical and surgical personnel and also for residents’ education especially concerning surgical training. Both the maintenance of oncological surgical activity, tending to implement extraordinary measures to limit contagion, and the management of all benign chronic and acute diseases of the colorectal and proctological spheres put a great strain on healthcare personnel (1). Despite this, some of the strategies implemented during the pandemic period showed surprising results under emergency conditions, which were also proposed afterwards with promising improvements in clinical practice. Among these, the use of telemedicine, but also the development of scores to stratify the population according to priority of need for treatment have been useful. In addition, new strategies of anesthesiologic and surgical approaches to limit infections have been formulated. ‘Awake surgery’ for example is a term borrowed from Neurosurgery, meaning major abdominal surgery performed on an awake patient in spontaneous breathing. During the coronavirus pandemic, the awake surgery technique was also introduced in general surgery. The advantages of awake surgery include reduced airway manipulation, reduced risk of infection in the operating room during the pandemic period, patient awake and spontaneously breathing during surgery, minimal nausea and vomiting, effective postoperative analgesia, early recovery after the surgical procedure, and reduced need for intensive care (Romanzi et al., Pietroletti et al.). Concerning the residency program however, a nationwide survey on the Italian scenario reported worrisome information on the training program of future surgeons during pandemic which deserves attention and planning of improvements to guarantee an adequate education (Gallo et al.). Those results were consistent with a previous survey concerning the first wave of the pandemic (2).

Surgery of the colon, rectum and anus, however, does not only concern neoplastic pathologies or major surgical treatments; in fact, in terms of frequency, benign surgery, proctology and functional pelvic floor disorders are by far the largest and sometimes, although benign, the most disabling. For example, about 40% of pregnant and post-partum women are affected by hemorrhoids and anal fissures, the treatment of which is often delayed for reasons of pregnancy or breastfeeding. These pathologies have such an impact on the patient's quality of life that they require the outmost attention for their prevention, management in the acute phase and their conservative or bridge-to-surgery treatment (Bužinskienė et al., Snopkova et al.).

In any case, anal fissure and proctological pathologies represent a challenging field for the surgeon, especially for the use of new products and technologies and also because of the heterogeneity in the choice of the correct surgical assessment (3). Giani et al. first proposed the Scanner-Assisted CO2 Laser Fissurectomy technique as a pilot study. Scanner-assisted CO2 laser showed great results in terms of pain control and wound healing, secondary to an extremely precise ablation, vaporization, and debridement procedures with minimal lateral thermal damage. Similarly, Alyanak et al. reported results on the comparison of botulinum toxin injection (BoNT) and left lateral sphincterotomy for the treatment of recurrent anal fissures, showing during the 3-month post-surgery follow-up period, that there was statistically significant difference (*p* < 0.01) between groups by pain and that neither technique was associated with deterioration in the incontinence scores during the 6-month post-surgery period. They therefore propose the traditional lateral internal sphincterotomy (LIS) technique as the most suitable and best in terms of pain and postoperative outcomes. Moreover, other authors investigated the complex treatment of perianal fistulas with preservation of the sphincter complex, concluding that the incidence rate of complications after fistulectomy treatment was higher than the others (*P* < 0.05) and that ligation of the intersphincteric fistula tract (imLIFT) may be the surgical method with the lowest incidence of postoperative complications (Huang et al.). Taking a step back towards the use of topical products for the conservative treatment of anal fissure, Tomasicchio et al. proposed the use of a topical gel for the treatment of the first uncomplicated presentation of fissure with an overall decrease in the VAS scale decreased significantly from 7 (IQR 4.7–8) at baseline to 1 (IQR 0–3.2, *p* = 0.05) after 20 days and a rate.

Promising results for the future have been published on the attempt of performing a pelvic floor transplant on rat models. Their microsurgical technique for pelvic floor transplantation in rats achieved an early survival rate of 81.82% that might open future scenarios on management of severe pelvic floor dysfunction with fecal and urinary incontinence, extensive perineal trauma, or congenital disorders (Galvao et al.).

However, especially for functional pathologies, there are multiple aspects ranging from conservative medical treatment, lifestyle, the gastroenterological aspect, pain management, surgical technique, the postoperative course, and the psychological aspect that all play a fundamental role in the successful management and treatment of the pathology that can be defined as multifactorial (4).

Hemorrhoidal prolapse is another benign extremely common condition that poses a treatment challenge to the surgeon. In recent years, several new techniques with increasingly stringent indications have been introduced. The challenge for the surgeon today is to find an algorithm to identify the best procedure for the patient's pathology and to acquire the necessary expertise to perform it best. Some studies have investigated the role and relevance of Goligher's classification, demonstrating the impossibility of providing today an adequate treatment algorithm. Consequently, it is important to emphasize the fundamental role of symptoms and clinical examination in assessing the pathology (5).

On this regard, numerous strategies have been implemented to study the best conservative treatment for early cases or bridge to surgery for more severe ones with the use of 3% polidocanol foam sclerotherapy or the better management of acute manifestations of hemorrhoidal prolapse such as hemorrhoid thrombosis. [Picciariello et al., Goglia et al., Lisi et al., Lobascio et al. (6, 7),] Even THD for hemorroidal prolapse reported to be another safe and reproducible technique, both as a first intervention and on recurrence. Physician and patient need to understand each other's expectations, weight the risks and benefits, and customize the treatment (Verre et al.). Anal stenosis after conventional treatment of hemorrhoidal disease has been investigated too, concluding that both complications and recurrence were significantly lower after house flap compared with rhomboid/diamond and Y-V flap (8–10).

Concerning the treatment of postoperative pain from haemorrhoidectomy, one of the well-established therapies is the subcutaneous injection of methylene blue (11). Studies on doses have been conducted concluding that the injection of 0.1% methylene blue has been shown to be equally effective at higher doses and safer (Long et al.).

However, medical management is pivotal in these patients both in the preoperative and in the postoperative phase. Beyond hemorrhoidal disease, a gastroenterological disorder associated with chronic constipation or obstructed defecation syndrome is frequently identified and it is usually responsible of the worsening of symptoms and the recurrency of the pathology after surgical treatment (Li et al.).

Moreover, psychological support is of paramount importance in proctological pathologies. In fact, the symptoms of such pathologies are frequently extremely disabling in daily activities and the patient feels ashamed when dealing with the doctor. The doctor-patient relationship is definitely to be improved with specialized communication techniques, but a clear association and need for psychological support has been demonstrated. Furthermore, certain psychiatric disorders have been associated with the specific recurrence of rectal prolapse as well as psychological support for postoperative pain management have been investigated. [Brochard et al., Wang et al. (12),]

The present editorial offered the opportunity to range from the hottest current topics in oncological medicine and surgery to minor but relevant adjustments. “Small major” changes are routinely done in major abdominal surgery. However, innovations and technologies are constantly growing and expanding, and the general surgeon must be ready to embrace and make the most of them in order to achieve the best outcome for patient care from the presentation of symptoms to the best and least painful and long-lasting postoperative care.
